# Drug combination choices of 5α-reductase inhibitors and α-blockers in patients with benign prostatic hyperplasia: a study based on the FAERS database

**DOI:** 10.3389/fmed.2026.1870053

**Published:** 2026-06-15

**Authors:** Jinqin Qian, Yanqing Gong, Cuijian Zhang, Liqun Zhou

**Affiliations:** 1 Department of Urology, Peking University First Hospital, Beijing, China; 2 The Institution of Urology, Peking University, Beijing, China; 3 Department of Urology, The First Affiliated Hospital of Henan University, Kaifeng, China

**Keywords:** 5α-reductase inhibitors, benign prostatic hyperplasia, drug combination therapy, FAERS database, α-blockers

## Abstract

**Background:**

In clinical practice, 5α-reductase inhibitors (5ARIs) and α-blockers (ABs) are commonly prescribed for benign prostatic hyperplasia (BPH). However, evidence supporting rational selection of combination therapy remains limited. The FDA Adverse Event Reporting System (FAERS) database is a valuable resource for investigating the safety profile and concomitant use patterns of these medications.

**Methods:**

This study analyzed adverse events (AEs) from 2004Q1 to 2024Q4 in BPH patients receiving combination therapy with 5ARIs and ABs. Drug–drug interactions (DDIs) were evaluated using five established algorithms, and disproportionality analysis was performed to identify targeted AEs associated with these medication classes.

**Results:**

Disproportionality analysis revealed that 5ARIs were predominantly linked to sexual dysfunction, whereas ABs were primarily associated with renal impairment. Additionally, certain drugs showed strong associations with physical discomfort, neurological symptoms, hypotension, and shock-related events. DDI analysis indicated that co-administration of 5ARIs and ABs may exacerbate both renal impairment and sexual dysfunction. Notably, the combination of tamsulosin and dutasteride significantly prolonged the time to resistance to dutasteride in BPH patients. These findings are consistent with prior clinical studies and align well with existing clinical and epidemiological evidence.

**Conclusion:**

The results provide meaningful insights into optimizing drug selection in BPH treatment. We recommend close monitoring of relevant safety parameters when implementing combination therapies.

## 1 Background

Benign prostatic hyperplasia (BPH), a highly prevalent urological disorder, mainly affects elderly men (1). With the global trend of population aging on the rise, BPH is placing an increasingly heavy medical and economic burden (2–4). Pathologically, BPH is marked by the progressive lesion of glandular and stromal tissues in the transition or periurethral zone of the prostate, usually presenting as progressive lower urinary tract symptoms (LUTS) (5). In recent years, pharmacological therapy has emerged as a central treatment approach for patients with benign prostatic hyperplasia (BPH) (6). Currently, 5ARIs and ABs are the most commonly prescribed medications in clinical practice. While combination therapy with these two drug classes appears to offer enhanced therapeutic benefits for BPH patients, there remains a paucity of studies addressing the optimal selection of drug combinations and their associated adverse effects (AEs) (7).

Evidence demonstrates that during the management of BPH, both 5ARIs and ABs exhibit dose-dependent associations with certain adverse events (8, 9). Combination therapy with these agents enables the attainment of comparable therapeutic outcomes while minimizing the need for high-dose monotherapy (10). This strategy may theoretically reduce the risk of AEs associated with elevated drug dosages. During combination therapy with the two drug classes, patients with BPH may experience AEs such as hypotension, sexual dysfunction, and fatigue (11–13). However, different drug combination regimens vary in terms of AEs. Therefore, conducting a comprehensive assessment of potential AEs in BPH patients concurrently using 5ARIs and ABs through large-scale post-marketing pharmacovigilance data is of critical importance.

The FDA Adverse Event Reporting System (FAERS) is one of the largest pharmacovigilance databases in the world, and conducts safety monitoring of drugs approved by the FDA by collecting spontaneous reporting data (14, 15). Analysis of large-scale reported data through systematic organization and data mining provides valuable insights into the real-world AEs experienced by BPH patients undergoing combination therapy (16). In this study, we systematically analyzed AEs of the target drugs from the first quarter of 2004 to the fourth quarter of 2024, with a specific focus on preferred terms (PTs) associated with combined medication use. This study aims to provide preliminary evidence regarding the safety and potential differences among various combinations of 5ARIs and ABs, offering clinicians valuable insights for rational drug selection and safety monitoring in combination therapy.

## 2 Methods

### 2.1 Data processing

In this study, we conducted a retrospective analysis of spontaneous reports from the FAERS database^1^ covering the period from the 2004Q1 to the 2024Q4. This work has been reported in line with the Standards for Quality Improvement Reporting Excellence (SQUIRE) criteria and TITAN criteria (17, 18). Comprehensive case safety reports encompassing AEs linked to medication utilization were gathered from patients diagnosed with BPH across the globe. The collected data from the FAERS database comprised seven standardized case report forms: demographic and administrative information (DEMO), drug information (DRUG), adverse events (REAC), patient outcomes (OUTC), report sources (RPSR), start and end dates of reported drug use (THER), and indications for use (INDI). According to the FDA-recommended method for removing duplicate reports, the PRIMARYID, CASEID, and FDADT fields in the DEMO table are selected and sorted in the order of CASEID, FDADT, and PRIMARYID. For reports with the same CASEID, the one with the largest FDADT value is retained. Secondly, for reports with the same CASEID and FDADT, the one with the largest PRIMARYID value is retained (19).

### 2.2 Identification of the drugs and AEs

In this study, we selected the 5ARIs (Dutasteride, Finasteride) and ABs (Alfuzosin, Doxazosin, Silodosin, Tamsulosin, Terazosin) most commonly utilized in clinical practice. To ensure comprehensive identification of drug names, we systematically retrieved both trade names and alternative designations for each medication using its corresponding Medical Subject Headings (MeSH) terms from the National Center for Biotechnology Information (NCBI) database. In this study, we incorporated all four types of drug roles in AEs—preferred suspect (PS), secondary suspect (SS), concomitant (C), and interacting (I)—to comprehensively assess potential DDIs among the medications under investigation.

Subsequently, based on the high-level group terms (HLGTs) within this hierarchical structure, we identified and selected PTs from the MedDRA (version 26.1) corresponding to clinically common complications (20). These PTs were categorized into five major groups: “Physical discomfort,” “Sexual dysfunction,” “Renal impairment,” “Neurological disorder,” and “Hypotension and shock.” A total of 103 target PTs across these five groups were included for the analysis of major AEs from the reporting odds ratio (ROR) and DDI analysis (Supplementary Table S1).

### 2.3 Signal detection and statistical analysis

In this study, we initially performed a disproportionality analysis to assess the potential association between the two drug classes administered to patients with BPH and the selected AEs, employing the ROR test. A 2 × 2 contingency table was constructed as the analytical framework for calculating the ROR (21) (Supplementary Tables S2, S3). Subsequently, to evaluate DDI signals, we organized the DDI signal data for analysis using a 4 × 2 contingency table to examine the presence or absence of AEs associated with each 5ARIs and each ABs (Supplementary Table S4). We applied five computational models described in the literature: (1) the *Ω* shrinkage model, (2) the additive model (Add), (3) the multiplicative model (Mul), (4) the chi-square model, (5) and the Combination Risk Ratio (CRR) model (22, 23) (Supplementary Table S5). The relevant data and statistical analyses were conducted using the corresponding R packages within the R environment (version 4.3.3).

## 3 Results

### 3.1 Data selection and baseline information

**Figure 1:**
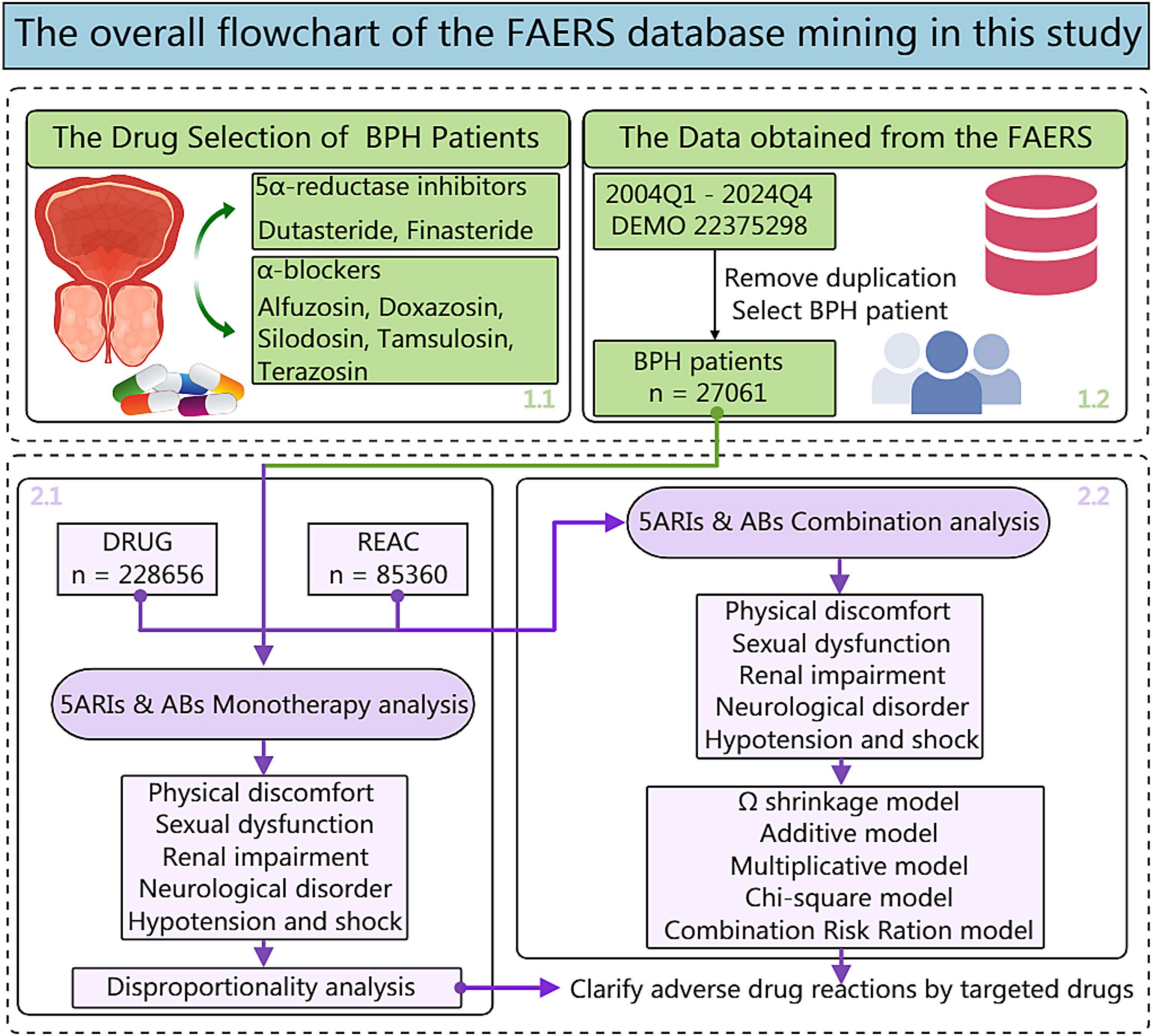
Flowchart of data extraction and deduplication from the FAERS database (2004Q1–2024Q4).

As shown in Figure 1, the spontaneous reports of BPH patients were extracted from the FAERS database for the period spanning 2004Q1 to 2024Q4. The initial dataset included 27,071 DEMO records, which was reduced to 27,061 after duplicate removal. A total of 228,656 DRUG entries were identified, comprising 18,048 records related to ABs as target drugs and 8,403 records involving 5ARIs as target drugs.

A statistical analysis was conducted on the baseline data of patients diagnosed with BPH who were administered 5ARIs or ABs, as presented in Table 1. There are a large number of missing values in the weight information, which is closely related to the defect of self-reporting. In terms of age, patients aged 65–85 years predominated in each drug group, while those under 18 years old are relatively rare, which is closely related to the age distribution of BPH patients. Regarding the severity of adverse reactions, patients using Dutasteride and Alfuzosin may be prone to severe complications, while the fatality rate in the Tamsulosin group is relatively high. In terms of occupation, the proportion of reports from consumers and doctors is relatively high.

**Table 1:** Baseline characteristics of patients with BPH receiving monotherapy with 5ARIs or ABs.

Variables	Dutasteride	Finasteride	Alfuzosin	Doxazosin	Silodosin	Tamsulosin	Terazosin
*n*	5,046	3,449	2,183	1,246	1,816	12,258	1,082
Weight (kg)							
<50	43 (0.9%)	23 (0.7%)	6 (0.3%)	11 (0.9%)	65 (3.6%)	178 (1.5%)	6 (0.6%)
>100	146 (2.9%)	267 (7.7%)	129 (5.9%)	75 (6.0%)	49 (2.7%)	641 (5.2%)	114 (10.5%)
50–100	1,239 (24.6%)	1,555 (45.1%)	850 (38.9%)	444 (35.6%)	784 (43.2%)	4,854 (39.6%)	441 (40.8%)
Missing	3,618 (71.7%)	1,604 (46.5%)	1,198 (54.9%)	716 (57.5%)	918 (50.6%)	6,585 (53.7%)	521 (48.2%)
Age (year)							
<18	31 (0.6%)	2 (0.1%)	1 (0.0%)	3 (0.2%)	14 (0.8%)	49 (0.4%)	0 (0.0%)
>85	407 (8.1%)	332 (9.6%)	220 (10.1%)	89 (7.1%)	172 (9.5%)	818 (6.7%)	83 (7.7%)
18–64.9	706 (14.0%)	488 (14.1%)	413 (18.9%)	217 (17.4%)	264 (14.5%)	2,084 (17.0%)	239 (22.1%)
65–85	2,891 (57.3%)	2,033 (58.9%)	1,322 (60.6%)	737 (59.1%)	1,128 (62.1%)	7,028 (57.3%)	596 (55.1%)
Missing	1,011 (20.0%)	594 (17.2%)	227 (10.4%)	200 (16.1%)	238 (13.1%)	2,279 (18.6%)	164 (15.2%)
Seruous							
No	2,775 (55.0%)	1,131 (32.8%)	717 (32.8%)	556 (44.6%)	402 (22.1%)	4,591 (37.5%)	681 (62.9%)
Yes	2,271 (45.0%)	2,318 (67.2%)	1,466 (67.2%)	690 (55.4%)	1,414 (77.9%)	7,667 (62.5%)	401 (37.1%)
Fatal							
No	4,796 (95.0%)	3,200 (92.8%)	1984 (90.9%)	1,160 (93.1%)	1,603 (88.3%)	11,406 (93.0%)	1,037 (95.8%)
Yes	250 (5.0%)	249 (7.2%)	199 (9.1%)	86 (6.9%)	213 (11.7%)	852 (7.0%)	45 (4.2%)
Reporter type							
Consumer	2,935 (58.2%)	1,147 (33.3%)	490 (22.4%)	455 (36.5%)	487 (26.8%)	4,135 (33.7%)	292 (27.0%)
Health Professional	133 (2.6%)	193 (5.6%)	117 (5.4%)	50 (4.0%)	118 (6.5%)	731 (6.0%)	23 (2.1%)
Pharmacist	338 (6.7%)	257 (7.5%)	275 (12.6%)	118 (9.5%)	184 (10.1%)	1,114 (9.1%)	332 (30.7%)
Physician	1,243 (24.6%)	1,224 (35.5%)	795 (36.4%)	379 (30.4%)	770 (42.4%)	4,432 (36.2%)	270 (25.0%)
Missing	397 (7.9%)	628 (18.2%)	506 (23.2%)	244 (19.6%)	257 (14.2%)	1846 (15.1%)	165 (15.2%)

### 3.2 ROR analysis for BPH patients

We assessed the specific AEs associated with monotherapy using 5ARIs or ABs in patient with BPH using the ROR method. For the individual use of 5ARIs, as shown in Table 2, both dutasteride (ROR: 1.81, 95% CI 1.57–2.07) and finasteride (ROR: 1.73, 95% CI 1.50–2.00) are associated with an increased risk of sexual dysfunction. Dutasteride (ROR: 1.11, 95% CI 1.01–1.23) may be associated with an increased risk of physical discomfort, whereas finasteride (ROR: 1.15, 95% CI 1.00–1.31) may be linked to a higher risk of renal impairment.

**Table 2:** The disproportionality analysis of each drugs for PT groups in BPH patients.

PT	Physical discomfort		Sexual dysfunction		Renal impairment		Neurological disorder		Hypotension and shock	
Drug	ROR(95%CI)	*X* ^2^	ROR(95%CI)	*X* ^2^	ROR(95%CI)	*X* ^2^	ROR(95%CI)	*X* ^2^	ROR(95%CI)	*X* ^2^
5ARIs										
Dutasteride	1.11(1.01–1.23)	4.57	1.81 (1.57–2.07)	71.99	0.67 (0.57–0.78)	27.24	0.72 (0.65–0.79)	46.86	0.48 (0.41–0.58)	69.77
Finasteride	1.01 (0.91–1.12)	0.01	1.73 (1.50–2.00)	56.98	1.15 (1.00–1.31)	4.13	0.66 (0.59–0.73)	66.60	0.66 (0.56–0.77)	26.21
ABs										
Alfuzosin	0.87 (0.75–1.01)	3.33	0.50 (0.37–0.68)	20.67	1.34 (1.13–1.58)	11.98	1.24 (1.12–1.39)	15.43	1.59 (1.36–1.86)	35.43
Doxazosin	1.11 (0.94–1.31)	1.56	0.72 (0.52–1.00)	3.82	1.78 (1.49–2.14)	39.88	1.03 (0.89–1.19)	0.14	1.23 (0.99–1.52)	3.67
Silodosin	0.83 (0.70–0.98)	4.87	1.45 (1.17–1.80)	11.82	0.95 (0.77–1.17)	0.20	0.95 (0.83–1.08)	0.67	0.87 (0.70–1.08)	1.50
Tamsulosin	1.13 (1.05–1.22)	10.36	0.78 (0.69–0.89)	15.23	1.23 (1.11–1.36)	16.69	1.14 (1.07–1.22)	16.72	1.05 (0.95–1.16)	1.02
Terazosin	1.14 (0.96–1.35)	2.09	0.65 (0.45–0.93)	5.62	1.33 (1.07–1.65)	6.70	2.58 (2.31–2.88)	306.52	3.58 (3.09–4.16)	324.00

For the individual use of ABs, tamsulosin (ROR: 1.13, 95% CI 1.05–1.22) is associated with an increased risk of physical discomfort, while silodosin (ROR: 1.45, 95% CI 1.17–1.80) is associated with a higher risk of sexual dysfunction. Regarding renal impairment, alfuzosin (ROR: 1.34, 95% CI 1.13–1.58), doxazosin (ROR: 1.78, 95% CI 1.49–2.14), tamsulosin (ROR: 1.23, 95% CI 1.11–1.36), and terazosin (ROR: 1.33, 95% CI 1.07–1.65) are all associated with elevated risk. Neurological disorders are also associated with alfuzosin (ROR: 1.24, 95% CI 1.12–1.39), tamsulosin (ROR: 1.14, 95% CI 1.07–1.22), and terazosin (ROR: 2.58, 95% CI 2.31–2.88). Additionally, hypotension and shock are linked to alfuzosin (ROR: 1.59, 95% CI 1.36–1.86) and terazosin (ROR: 3.58, 95% CI 3.09–4.16).

### 3.3 DDI analysis for AEs among drug combinations

Subsequently, we conducted a DDI analysis to investigate the impact of drug combinations on specific categories of AEs. As shown in the Tables 3, 4. In terms of physical discomfort, the combination of tamsulosin with dutasteride (Mul: 1.05) or finasteride (Add: 0.01) is associated with an increased potential risk. For patients receiving dutasteride, concomitant use of tamsulosin may further exacerbate physical discomfort. Regarding sexual dysfunction, the co-administration of 5ARIs and ABs is associated with a higher risk. Specifically, dutasteride showed positive signals when combined with five ABs: alfuzosin (Mul: 12.36), doxazosin (Mul: 15.22), silodosin (Mul: 3.65), tamsulosin (Mul: 65.56), and terazosin (Add > 0 and Mul: 28.78). Notably, the dutasteride-terazosin combination yielded positive results in both algorithms, suggesting a stronger association with risk. Similarly, finasteride exhibited positive signals when used concomitantly with the same five ABs: alfuzosin (Mul: 19.64), doxazosin (Mul: 25.32), silodosin (Mul: 6.04), tamsulosin (Mul: 38.46), and terazosin (Mul: 46.02).

**Table 3:** Signal detection for various dutasteride-ABs combinations and targeted AEs.

DDI combination	*n*_111_	*n*_110_	*n*_11+_	Ω_025_	Additive	Multiplicative	Chi-square	CRR
Physical discomfort								
Dutasteride + Alfuzosin	314	8,764	9,078	−0.41	−0.01	0.90	−3.15	0.87
Dutasteride + Doxazosin	302	8,326	8,628	−0.49	−0.01	0.86	−3.92	0.83
Dutasteride + Silodosin	305	8,496	8,801	−0.37	−0.01	0.96	−2.54	0.91
Dutasteride + Tamsulosin	671	18,296	18,967	−0.18	0.00	1.05	−1.35	1.00
Dutasteride + Terazosin	290	8,102	8,392	−0.44	−0.01	0.88	−3.28	0.85
Sexual dysfunction								
Dutasteride + Alfuzosin	257	8,821	9,078	−0.44	−0.01	12.36	−3.01	0.87
Dutasteride + Doxazosin	251	8,377	8,628	−0.32	0.00	15.22	−1.54	0.92
Dutasteride + Silodosin	310	8,491	8,801	−1.25	−0.04	3.65	−13.62	0.85
Dutasteride + Tamsulosin	545	18,422	18,967	−1.10	−0.03	65.56	−16.24	1.25
Dutasteride + Terazosin	246	8,146	8,392	−0.34	0.00	28.78	−1.77	0.89
Renal impairment								
Dutasteride + Alfuzosin	53	9,025	9,078	−2.27	0.01	0.10	−10.31	0.28
Dutasteride + Doxazosin	46	8,582	8,628	−2.43	0.01	0.09	−10.35	0.26
Dutasteride + Silodosin	43	8,758	8,801	−2.58	0.01	0.12	−10.79	0.24
Dutasteride + Tamsulosin	100	18,867	18,967	−2.67	0.02	0.15	−18.59	0.24
Dutasteride + Terazosin	38	8,354	8,392	−2.71	0.02	0.15	−10.73	0.23
Neurological disorder								
Dutasteride + Alfuzosin	378	8,700	9,078	−1.41	−0.04	0.24	−17.68	0.40
Dutasteride + Doxazosin	315	8,313	8,628	−1.39	−0.03	0.24	−15.62	0.41
Dutasteride + Silodosin	337	8,464	8,801	−1.42	−0.03	0.24	−16.68	0.40
Dutasteride + Tamsulosin	1,232	17,735	18,967	−0.52	−0.02	0.57	−10.83	0.72
Dutasteride + Terazosin	472	7,920	8,392	−2.38	−0.20	0.13	−37.54	0.20
Hypotension and shock								
Dutasteride + Alfuzosin	122	8,956	9,078	−2.09	−0.02	0.08	−15.06	0.26
Dutasteride + Doxazosin	97	8,531	8,628	−2.25	−0.02	0.07	−14.56	0.24
Dutasteride + Silodosin	73	8,728	8,801	−1.74	0.00	0.10	−8.75	0.35
Dutasteride + Tamsulosin	426	18,541	18,967	−0.73	0.00	0.19	−8.52	0.62
Dutasteride + Terazosin	203	8,189	8,392	−2.95	−0.13	0.05	−31.48	0.14

**Table 4:** Signal detection for various finasteride-ABs combinations and targeted AEs.

DDI combination	*n*_111_	*n*_110_	*n*_11+_	Ω_025_	Additive	Multiplicative	Chi-square	CRR
Physical discomfort								
Finasteride + Alfuzosin	193	6,235	6,428	−0.58	0.00	0.62	−3.70	0.76
Finasteride + Doxazosin	181	5,797	5,978	−0.72	−0.01	0.58	−4.80	0.69
Finasteride + Silodosin	184	5,967	6,151	−0.60	0.00	0.62	−3.73	0.76
Finasteride + Tamsulosin	550	15,767	16,317	−0.24	0.01	0.65	−1.91	0.91
Finasteride + Terazosin	169	5,573	5,742	−0.67	0.00	0.58	−4.09	0.72
Sexual dysfunction								
Finasteride + Alfuzosin	226	6,202	6,428	−0.56	−0.01	19.64	−3.97	0.79
Finasteride + Doxazosin	220	5,758	5,978	−0.46	−0.01	25.32	−2.83	0.83
Finasteride + Silodosin	279	5,872	6,151	−1.06	−0.04	6.04	−10.46	1.10
Finasteride + Tamsulosin	514	15,803	16,317	−1.28	−0.04	38.46	−18.64	0.92
Finasteride + Terazosin	215	5,527	5,742	−0.43	0.00	46.02	−2.40	0.85
Renal impairment								
Finasteride + Alfuzosin	41	6,387	6,428	−2.12	0.01	0.12	−7.97	0.32
Finasteride + Doxazosin	34	5,944	5,978	−2.32	0.01	0.09	−8.05	0.28
Finasteride + Silodosin	31	6,120	6,151	−2.53	0.01	0.13	−8.57	0.25
Finasteride + Tamsulosin	88	16,229	16,317	−2.55	0.02	0.18	−16.23	0.25
Finasteride + Terazosin	26	5,716	5,742	−2.71	0.01	0.15	−8.51	0.23
Neurological disorder								
Finasteride + Alfuzosin	277	6,151	6,428	−1.37	−0.03	0.20	−14.32	0.42
Finasteride + Doxazosin	214	5,764	5,978	−1.47	−0.03	0.20	−13.44	0.40
Finasteride + Silodosin	236	5,915	6,151	−1.46	−0.03	0.19	−14.08	0.40
Finasteride + Tamsulosin	1,131	15,186	16,317	−0.45	−0.01	0.44	−8.68	0.76
Finasteride + Terazosin	371	5,371	5,742	−2.22	−0.19	0.12	−30.21	0.23
Hypotension and shock								
Finasteride + Alfuzosin	114	6,314	6,428	−1.64	−0.02	0.14	−10.63	0.38
Finasteride + Doxazosin	89	5,889	5,978	−1.77	−0.01	0.12	−10.09	0.35
Finasteride + Silodosin	65	6,086	6,151	−1.39	0.00	0.18	−6.01	0.47
Finasteride + Tamsulosin	418	15,899	16,317	−0.50	0.00	0.37	−5.17	0.76
Finasteride + Terazosin	195	5,547	5,742	−2.43	−0.12	0.09	−23.85	0.21

With regard to renal impairment, the combination of these two drug classes increases the risk of kidney injury. Positive signals were observed for dutasteride in combination with all five ABs (alfuzosin, Add: 0.01; doxazosin, Add: 0.01; silodosin, Add: 0.01; tamsulosin, Add: 0.02; terazosin, Add: 0.02), as well as for finasteride (alfuzosin, Add: 0.01; doxazosin, Add: 0.01; silodosin, Add: 0.01; tamsulosin, Add: 0.02; terazosin, Add: 0.01). In contrast, regarding neurological disorders, the combination does not appear to elevate the associated risks and may instead reflect idiopathic symptoms related to certain ABs. Concerning hypotension and shock, the combination of dutasteride and silodosin (Add > 0) is associated with an increased risk, whereas finasteride combined with either silodosin (Add > 0) or tamsulosin (Add > 0) also confers an elevated risk.

### 3.4 Analysis of cumulative risk curves for weakened drug efficacy

**Figure 2:**
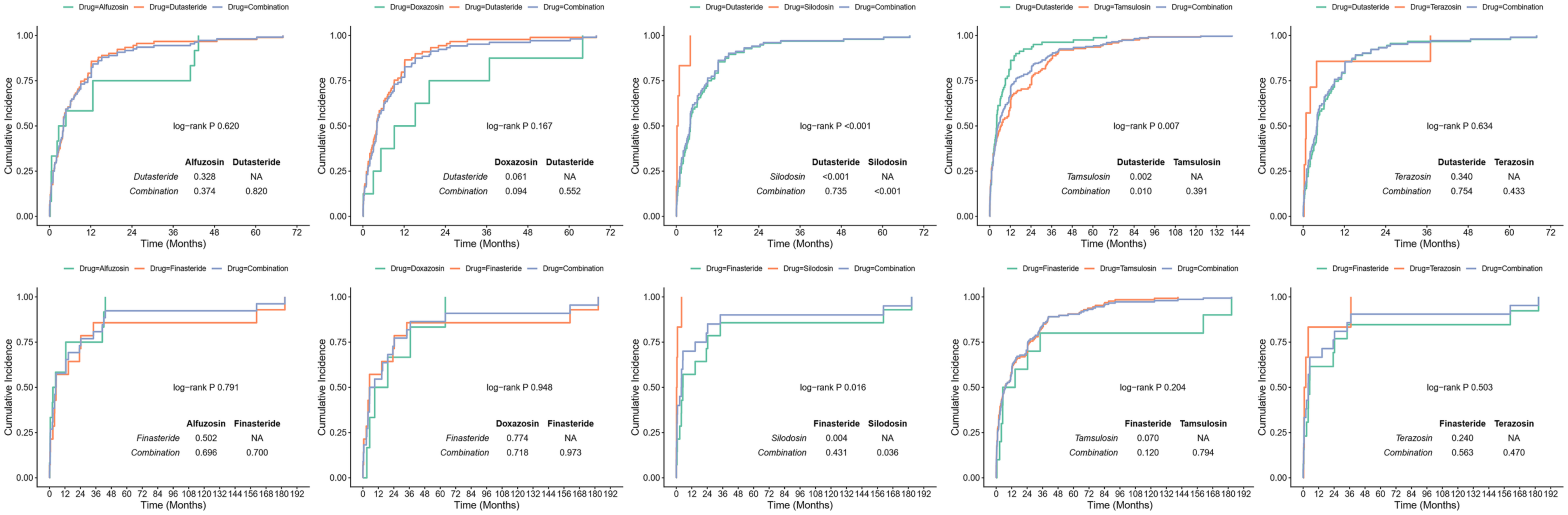
Cumulative risk curves for reduced drug efficacy in monotherapy versus combination therapy. The *x*-axis represents the duration of medication, and the *y*-axis represents the cumulative risk curve.

We collected AEs related to the decline in drug efficacy as PTs and FAERS case data containing follow-up times. As shown in Figure 2, when comparing the time to develop drug resistance between the combination use of two drugs and the use of a single drug, the combination of dutasteride + silodosin, dutasteride + tamsulosin, and finasteride + silodosin demonstrated less drug resistance with the two-drug combination. Among them, due to the relatively small amount of data on silodosin, the data on the combination of silodosin and 5ARIs may not well indicate that it can reduce the risk of silodosin monotherapy resistance. The most obvious is the combination of dutasteride and tamsulosin, which can effectively reduce the risk of resistance to dutasteride monotherapy (*p* = 0.002).

## 4 Discussion

Over the past decades, the proportion of patients with BPH receiving pharmacological treatment has increased (24–26). Although BPH itself is not always a direct indication for intervention, the associated LUTS significantly impair patients’ quality of life. In clinical practice, given the abundance of *α*-adrenergic receptor-regulated smooth muscle in the prostate and its surrounding tissues, α-adrenergic receptor blockers are widely used to alleviate urinary symptoms by reducing urethral resistance. Furthermore, BPH progression is closely linked to dihydrotestosterone (DHT), a potent androgen derived from testosterone via the action of 5α-reductase. Elevated DHT levels promote prostate growth, contributing to glandular enlargement and subsequent urethral compression. By inhibiting 5α-reductase, DHT synthesis is suppressed, leading to a reduction in prostate volume and long-term improvement in LUTS. Therefore, targeting both α-adrenergic receptors and 5α-reductase represents a dual therapeutic strategy that addresses both symptom relief and disease modification in BPH management. Meanwhile, the proportion of BPH patients treated with combination therapy has increased significantly (26). However, there is currently a lack of evidence to support rational decision-making regarding the combined use of 5ARIs and ABs. Therefore, mining real-world spontaneous reporting data is essential.

In this study, we selected the most common AEs observed in patients with BPH for analysis and categorized them into five AE groups based on MedDRA terminology. From the FAERS database, we identified case reports associated with patients diagnosed with BPH and calculated the ROR for relevant AE groups during the use of 5ARIs and ABs using disproportionality analysis. Among the overall baseline data, missing values are predominantly observed for weight and age. This is unlikely to substantially affect the ROR analysis.

We further examined the presence of positive signals for individual PTs under monotherapy conditions. In the monotherapy analysis, the impact of 5ARIs on sexual dysfunction was significant, consistent with the results reported in the literature (27, 28). Among them, Dutasteride was more likely to cause physical condition, while Finasteride might be associated with renal impairment. The main AEs of ABs included renal impairment, neurological disorders, hypotension and shock, which had high clinical warning significance. In contrast, Silodosin did not show the above AEs, but there was a certain association between it and sexual dysfunction. Previous studies have also confirmed the impact of silodosin on sexual dysfunction, which may be closely related to its role as a selective ABs (29).

Subsequently, five DDI analyses methods were applied to assess potential DDI signals between 5ARIs and ABs as described in previous literature (22, 30). Various statistical approaches have been established to identify DDI signals. However, no universally accepted gold standard currently exists for signal detection in spontaneous reporting systems, because each method has unique strengths and weaknesses (31, 32). To establish a comprehensive screening framework, our study combined five distinct statistical algorithms: the additive model, the multiplicative model, the combination risk ratio (CRR) model, the chi-square model, and the *Ω* shrinkage measure, which are among the most commonly used frequency-based approaches specifically designed or adapted for DDI signal detection in spontaneous reporting databases. These methods were chosen because they provide complementary perspectives on potential DDIs and exhibit different trade-offs between sensitivity and specificity (22). According to previous comparative evaluations by Noguchi et al., the *Ω* shrinkage measure is relatively conservative and demonstrated the highest specificity among the evaluated models (22). In contrast, the additive model tends to identify a larger number of potential signals, suggesting higher sensitivity for initial signal screening but lower specificity. Conversely, when sufficient report counts are available (*n*₁₁₁ ≥ 3), the chi-square and CRR models display substantially higher negative agreement rates when compared to the Ω model, indicating improved false-positive control and higher specificity; the multiplicative model maintains a moderate balance between sensitivity and specificity (22). By integrating multiple algorithms with different operating characteristics, our study aimed to achieve complementary evaluation of potential DDI signals while reducing reliance on any single statistical approach. Signals consistently identified across multiple methods may therefore be considered more robust and less likely to represent false-positive associations, thereby improving the reliability of candidate signals for subsequent clinical assessment.

In recent years, combination therapy for BPH has become a focal point of clinical research, reflecting growing interest in optimizing therapeutic efficacy (33, 34). Parallel to these developments, increasing attention has been directed toward the assessment of adverse effects associated with combination regimens, particularly sexual dysfunction, to better inform risk–benefit profiles and guide clinical decision-making (35, 36). This study also found that the combined use of 5ARIs and ABs can increase the risk of sexual dysfunction and renal impairment. Among them, the combination of silodosin and 5ARIs may further increase the risk of hypotension and shock, while the combination of tamsulosin and 5ARIs may exacerbate the occurrence of physical discomfort. Therefore, for patients with underlying diseases, the potential adverse events caused by combined medication deserve high attention. Meanwhile, we also collected the relevant PTs of reduced or ineffective drug response and conducted related analyses. We found that the combined use of tamsulosin and dutasteride can prolong the time window of dutasteride resistance. Previous literature rarely discusses drug resistance to dutasteride during the treatment of BPH. However, when tamsulosin is used in combination with dutasteride, the delay in the emergence of dutasteride resistance may be due to the reduction of the single-drug dose. Our findings are consistent with the CombAT study, which verified the clinical value of combined dutasteride and tamsulosin in BPH patients (34). The present analysis further supplements the safety characteristics of this regimen and supports its rational use in clinical practice.

All analyzed data in this study were sourced from the FAERS database, and the following limitations must be acknowledged. First, as a spontaneous reporting system, the FAERS database may involve uncorrectable underreporting, data bias, and duplicate entries. Additionally, the absence of denominator data limits the ability to calculate the true incidence of the related AEs among BPH patients. Second, this study relied on current pharmacovigilance methods to detect signal associations, which do not establish direct causality between drugs and adverse reactions. Third, the analysis primarily focused on combinations of two drugs, whereas real-world clinical practice may involve more complex polypharmacy regimens. Furthermore, potential confounding factors such as age and dosage could not be fully controlled. Despite these limitations, this study conducted a systematic evaluation based on large-scale real-world data, offering valuable insights into the rational use of medications for patients with BPH. Further validation through clinical cohort studies is warranted.

## 5 Conclusion

In conclusion, this study comprehensively investigated the potential AEs and drug interactions associated with the clinical use of two commonly prescribed 5ARIs and five ABs. With respect to sexual function impairment and renal impairment, combination therapy involving these drug classes is more likely to elevate associated risks. The findings of this study provide valuable insights for the rational selection of pharmacological regimens in patients with BPH.

## Data Availability

The original contributions presented in the study are included in the article/Supplementary material, further inquiries can be directed to the corresponding authors.

## Footnotes

1
https://fis.fda.gov/extensions/FPD-QDE-FAERS/FPD-QDE-FAERS.html
