# Supplementary material for: Drug combination choices of 5α-reductase inhibitors and α-blockers in patients with benign prostatic hyperplasia: a study based on the FAERS database
Source: Front Med (Lausanne). 2026 Jun 15;13:1870053. doi: 10.3389/fmed.2026.1870053 (PMC13310751; doi:10.3389/fmed.2026.1870053)
Supplement: Supplementary file 1 [file Table_1.docx]

**Appendix**

| **Table S1. The selected PTs in the five AE Groups** | | | | | |
| --- | --- | --- | --- | --- | --- |
| Physical discomfort | Sexual dysfunction | Renal impairment | Renal impairment | Hypotension and shock |  |
| FATIGUE | SPONTANEOUS PENILE ERECTION | ACUTE KIDNEY INJURY | UNRESPONSIVE TO STIMULI | ORTHOSTATIC HYPOTENSION |  |
| ASTHENIA | SPONTANEOUS EJACULATION | CHRONIC KIDNEY DISEASE | CEREBROSPINAL FLUID LEAKAGE | HYPOTENSIVE CRISIS |  |
| MALAISE | RETROGRADE EJACULATION | SUBACUTE KIDNEY INJURY | INVOLUNTARY VOCALISATION | HYPOTENSION |  |
| SLUGGISHNESS | PRIAPISM | SCLERODERMA RENAL CRISIS | TONGUE BITING | DIASTOLIC HYPOTENSION |  |
| DECREASED ACTIVITY | PAINFUL ERECTION | RENAL INJURY | SLOW RESPONSE TO STIMULI | CAPILLARY LEAK SYNDROME |  |
| CHRONIC FATIGUE SYNDROME | PAINFUL EJACULATION | RENAL IMPAIRMENT NEONATAL | PRESYNCOPE | SHOCK SYMPTOM |  |
| DISCOMFORT | ORGANIC ERECTILE DYSFUNCTION | RENAL IMPAIRMENT | PERSISTENT POSTURAL-PERCEPTUAL DIZZINESS | SHOCK HAEMORRHAGIC |  |
| PAIN | NOCTURNAL EMISSION | RENAL FAILURE NEONATAL | NEUROLOGICAL SYMPTOM | SHOCK |  |
| VISCERAL PAIN | ERECTION INCREASED | RENAL FAILURE | NEUROLOGICAL DECOMPENSATION | PERIPHERAL CIRCULATORY FAILURE |  |
| TENDERNESS | ERECTILE DYSFUNCTION | PRERENAL FAILURE | NEUROGLYCOPENIA | HYPOVOLAEMIC SHOCK |  |
| SUPRAPUBIC PAIN | EJACULATION FAILURE | POSTRENAL FAILURE | MYOCLONUS | HYPOPERFUSION |  |
| SUPRACLAVICULAR FOSSA PAIN | EJACULATION DISORDER | PANCREATORENAL SYNDROME | MENINGISM | DISTRIBUTIVE SHOCK |  |
| PSEUDOANGINA | EJACULATION DELAYED | OLIGURIA | INTRACRANIAL HYPOTENSION | CIRCULATORY COLLAPSE |  |
| PARADOXICAL PAIN |  | NEONATAL ANURIA | HEAD DISCOMFORT |  |  |
| NYCTALGIA |  | FOETAL RENAL IMPAIRMENT | DROOLING |  |  |
| NON-CARDIAC CHEST PAIN |  | END STAGE RENAL DISEASE | DIZZINESS POSTURAL |  |  |
| MAXILLOFACIAL PAIN |  | DIABETIC END STAGE RENAL DISEASE | DIZZINESS EXERTIONAL |  |  |
| FACIAL PAIN |  | CRUSH SYNDROME | DIZZINESS |  |  |
| FACIAL DISCOMFORT |  | ANURIA | CLONUS |  |  |
| CHEST PAIN |  |  | BRAIN FOG |  |  |
| CHEST DISCOMFORT |  |  | SYNCOPE |  |  |
| BREAKTHROUGH PAIN |  |  | STUPOR |  |  |
| AXILLARY PAIN |  |  | SOMNOLENCE |  |  |
|  |  |  | SEDATION |  |  |
|  |  |  | PREICTAL STATE |  |  |
|  |  |  | POSTICTAL STATE |  |  |
|  |  |  | LOSS OF CONSCIOUSNESS |  |  |
|  |  |  | LETHARGY |  |  |
|  |  |  | HYPOGLYCAEMIC UNCONSCIOUSNESS |  |  |
|  |  |  | HYPERGLYCAEMIC UNCONSCIOUSNESS |  |  |
|  |  |  | DEPRESSED LEVEL OF CONSCIOUSNESS |  |  |
|  |  |  | CONSCIOUSNESS FLUCTUATING |  |  |
|  |  |  | ALTERED STATE OF CONSCIOUSNESS |  |  |
|  |  |  | DISORIENTATION |  |  |
|  |  |  | CONFUSIONAL STATE |  |  |

**Table S2. The 2×2 contingency table for signal detection for disproportionality analysis**

|  | Target adverse events reported | Other adverse events reported | Total |
| --- | --- | --- | --- |
| Target drugs | $\text{a}$ | $\text{b}$ | a+b |
| Other drugs | $\text{c}$ | $\text{d}$ | c+d |
| Total | a+c | b+d | a+b+c+d |

**Table S3. The signal formula and detective criteria for disproportionality analysis**

| Method | Calculation formula | ﻿Criteria |
| --- | --- | --- |
| ROR | $\text{ROR=}\frac{\text{a / c}}{\text{b / d}}$ | a ≥ 3  95%CI (lower limit) > 1 |
|  | $\text{SE(}\text{lnROR}\text{)=}\sqrt{\frac{\text{1}}{\text{a}}\text{+}\frac{\text{1}}{\text{b}}\text{+}\frac{\text{1}}{\text{c}}\text{+}\frac{\text{1}}{\text{d}}}$ |  |
|  | $\text{95\%CI= }\text{e}^{\ln\left( \text{ROR} \right)\text{±1.96SE}}$ |  |
| PRR | $\text{PRR=}\frac{\text{a / (}\text{a+b}\text{)}}{\text{c / (}\text{c+d}\text{)}}$ | a ≥ 3  PRR ≥ 2  95%CI (lower limit) > 1 |
|  | $\text{SE(}\text{lnPRR}\text{)=}\sqrt{\frac{\text{1}}{\text{a}}\text{-}\frac{\text{1}}{\text{a+b}}\text{+}\frac{\text{1}}{\text{c}}\text{-}\frac{\text{1}}{\text{c+d}}}$ |  |
|  | $\text{95\%CI= }\text{e}^{\ln\left( \text{PRR} \right)\text{±1.96se}}$ |  |

**Table S4. The 4×2 contingency table for signal detection for disproportionality analysis**

|  | Target adverse events reported | Other adverse events reported | Total |
| --- | --- | --- | --- |
| Concomitant use of 5ARIs and ABs | $\text{n}\text{111}$ | $\text{n}\text{110}$ | n_11+_ |
| 5ARIs without ABs | $\text{n}\text{101}$ | $\text{n}\text{100}$ | n_10+_ |
| ABs without 5ARIs | n_011_ | n_010_ | n_01+_ |
| Neither 5ARIs nor ABs | n_001_ | n_000_ | n_00+_ |
| Total | n_++1_ | n_++0_ | _n+++_ |

**Table S5. Signal calculation formula and detection criteria for each DDI model**

| The DDI models | Calculation formula | Criteria for positive signals |
| --- | --- | --- |
| Ω shrinkage model | $\text{Ω}_{\text{025}}\text{ }\text{= Ω-}\frac{\text{ϕ(0.975)}}{\log\left( \text{2} \right)\sqrt{\text{n}_{\text{111}}}}$ | n111≥3, Ω025>0 |
| Additive model | Add = $\frac{\text{n}_{\text{111}}}{\text{n}_{\text{11+}}}\text{-}\frac{\text{n}_{\text{101}}}{\text{n}_{\text{10+}}}\text{-}\frac{\text{n}_{\text{011}}}{\text{n}_{\text{01+}}}\text{-}\frac{\text{n}_{\text{001}}}{\text{n}_{\text{00+}}}$ | n111≥3, Add>0 |
| Multiplicative model | Mul = $\frac{\text{n}_{\text{111}}\text{×}\text{n}_{\text{001}}\text{×}\text{n}_{\text{10+}}\text{×}\text{n}_{\text{01+}}}{\text{n}_{\text{11+}}\text{×}\text{n}_{\text{00+}}\text{×}\text{n}_{\text{101}}\text{×}\text{n}_{\text{011}}}$ | n111≥3, Mul>0 |
| Chi-square model | $\chi=\frac{n_{111}-E_{111}-0.5}{\sqrt{E_{111}}}$ | n111≥3, $\chi$>2 |
| Combination risk ratio model | $\text{CRR=}\frac{\text{PR}\text{R}_{\text{Drug5ARIs∩DrugABs}}}{\max\text{(}\text{PR}\text{R}_{\text{Drug5ARIs}}\text{,PR}\text{R}_{\text{DrugABs}}\text{)}}$ | n111≥3, PRR>2, X2>4, CRR>2 |
